# Association between serum zinc and copper concentrations and copper/zinc ratio with the prevalence of knee chondrocalcinosis: a cross-sectional study

**DOI:** 10.1186/s12891-020-3121-z

**Published:** 2020-02-12

**Authors:** Hongyi He, Yilun Wang, Zidan Yang, Xiang Ding, Tuo Yang, Guanghua Lei, Hui Li, Dongxing Xie

**Affiliations:** 1 Department of Orthopaedics, Xiangya Hospital, Central South University, #87 Xiangya Road, Changsha, 410008 Hunan Province China; 2 Department of Epidemiology and Health Statistics, Xiangya School of Public Health, Central South University, Changsha, Hunan China; 3 Health Management Center, Xiangya Hospital, Central South University, Changsha, Hunan China; 4 Hunan Engineering Research Center of Osteoarthritis, Changsha, Hunan China; 5 Hunan Key Laboratory of Joint Degeneration and Injury, Changsha, Hunan China; 6 National Clinical Research Center of Geriatric Disorders, Xiangya Hospital, Central South University, Changsha, Hunan China

**Keywords:** Zinc, Copper, Chondrocalcinosis

## Abstract

**Background:**

Patients with chondrocalcinosis may suffer from a series of symptoms resembling acute gouty arthritis or septic arthritis, but the aetiology and pathogenesis of chondrocalcinosis have not been fully elucidated yet. This study was aimed to assess serum zinc and copper concentrations, as well as the ratio of serum copper to zinc concentrations (Cu/Zn ratio), in relation to the prevalence of knee chondrocalcinosis.

**Methods:**

Data included in this analysis were retrieved from a large population-based cross-sectional study. A bilateral knee anteroposterior radiograph was obtained from each subject. Radiographic knee chondrocalcinosis was diagnosed if definite linear cartilage calcification was detected. Serum zinc and copper concentrations were measured using the spectrophotometric flow injection methods by Roche modular P800. The relations of serum zinc and copper concentrations and Cu/Zn ratio to the prevalence of knee chondrocalcinosis were examined using generalized estimating equations, respectively.

**Results:**

The prevalence of knee chondrocalcinosis was 1.2% in the sample of this study (*n* = 12,362). In comparison with the lowest tertile, the odds ratios (ORs) of knee chondrocalcinosis adjusted by age, sex and body mass index were 0.74 (95% CI 0.50–1.09) in the second and 0.56 (95% CI 0.36–0.86) in the third tertiles of serum zinc concentrations (*P* for trend = 0.009), were 1.26 (95% CI 0.77–2.05) in the second and 2.01 (95% CI 1.25–3.24) in the third tertile of serum copper concentrations (*P* for trend = 0.003), and were 1.02 (95% CI 0.61–1.69) in the second and 2.23 (95% CI 1.38–3.59) in the third tertile of Cu/Zn ratio (*P* for trend < 0.001) respectively. These findings were not materially altered by adjustment for potential confounders.

**Conclusions:**

The present study observed that higher serum zinc concentrations, lower serum copper concentrations or lower Cu/Zn ratio are associated with a lower prevalence of knee chondrocalcinosis in a dose-response relationship manner.

## Background

Chondrocalcinosis is a disease characterized by the formation of calcium pyrophosphate and basic calcium phosphate crystals in the pericellular matrix of cartilage, and by the calcification of articular fibrocartilage and hyaline cartilage [1–3]. Patients with chondrocalcinosis may suffer from fever, joint pain and a series of other clinical symptoms that are similar to acute gouty arthritis or septic arthritis and chronic symptoms resembling osteoarthritis, which all contributes to a compromised quality of life and a worsened comorbidity [4–8]. As for asymptomatic patients, though no clinical symptoms manifested, the crystals deposited on the cartilage surface may cause or accelerate joint damage by activating inflammatory response, altering the mechanical stress distribution on the cartilage surface and acting as wear particles, which was postulated as a condition representing a presymptomatic phase of clinical arthritis [9, 10]. However, the aetiology and pathogenesis of chondrocalcinosis have not been fully elucidated.

As important trace elements in the human body, zinc and copper are both involved in many homeostatic mechanisms, including immunity and inflammation [11–13]. While zinc was believed to be an anti-inflammatory factor, copper was suggested to be a proinflammatory agent [13, 14]. Previous studies have reported that many common diseases, such as cardiovascular disease, cancer, type 2 diabetes, Alzheimer’s disease, vitiligo and multiple sclerosis were associated with lower zinc concentrations and higher copper concentrations, of which the underlying mechanisms were suspected to be attributed to immune function, inflammatory response and oxidative stress [15–20]. In addition, not only is the individual concentrations of serum copper and zinc of concern, but the balance between these two elements appears to be important. Previous studies suggested that calculating the ratio of serum copper to zinc concentrations, hereafter referred to as Cu/Zn ratio, provided valuable information in regard to oxidative stress and inflammatory responses [21]. Due to the essential role of inflammatory response in the pathogenesis of chondrocalcinosis [22, 23], it was postulated that there might be a potential link between the serum zinc and copper concentrations, as well as Cu/Zn ratio, and the prevalence of chondrocalcinosis. However, to our best knowledge, no study has been performed to investigate these correlations yet.

To fill in the knowledge gap, data were collected from a large population-based cross-sectional study and used to examine the relationship between serum zinc and copper concentrations and Cu/Zn ratio with the prevalence of knee chondrocalcinosis so as to determine the shape of the corresponding dose-response relationship if it exists.

## Methods

### Study population

The subjects of this cross-sectional study were individuals from the general population who had undergone routine health examinations at Xiangya Hospital, Central South University in Changsha, Hunan, China, between October 2013 and December 2015. Identical study designs have already been reported in some previous publications [24–26]. Briefly speaking, the routine health checkups include anthropometric measurements (such as height and body mass), basic clinical tests (such as blood pressure and heart rate), biochemistry (such as blood tests, liver function, kidney function and trace element tests) and imaging tests (such as chest radiography and weight-bearing bilateral anterior-posterior knee radiography). The subjects qualified for this study were individuals satisfying the following inclusion criteria: (1) age ≥ 40 years; (2) completed the serum zinc and serum copper tests; (3) provided bilateral weight-bearing anteroposterior and bilateral axial knee X-ray films. Prior to the start of the study, all the involved interviewers, clinical inspectors, and X-ray technicians had been properly trained by the leading researchers.

During the study period, 31,542 participants underwent routine health checkups at the aforementioned study center and 12,420 of them met all the inclusion criteria. Then, 58 participants were eliminated according to the exclusion criteria: (1) the weight-bearing anteroposterior knee radiograph was in unsatisfactory quality (*n* = 53); and (2) the Kellgren-Lawrence (KL) [27] grades of both knees were 4 (*n* = 5). Eventually, 12,362 participants were included in the final analysis.

### Blood biochemical analysis

The venous blood sample was collected from each subject upon an overnight fast of 12 h with a serum separation tube (containing silica clot activators and gel separator) and was immediately centrifuged at 4 °C for 10 min at 3000 relative centrifugal force. The serum zinc and serum copper concentrations were detected by the spectrophotometric flow injection methods, “2-(5-Bromo-2-pyridylazo)-5-[N-propyl-N-(3-sulfopropyl) amino] phenol”, i.e., 5-Br-PAPS, and the “4-(3,5-dibromo-2-pyridylazo)-N-ethyl-N-(3-sulfopropyl) aniline”, i.e., 3,5-diBr-PAESA, respectively, using Roche modular P800 [28–30].

The inter-assay coefficients of variation were 4.44% (10.1 μmol/L) and 2.50% (18.5 μmol/L) and the intra-assay coefficients of variation were 2.86% (18.74 μmol/L) and 3.42% (9.49 μmol/L) for serum zinc. Likewise, the inter-assay coefficients of variation were 6.15% (9.8 μmol/L) and 4.33% (16.5 μmol/L), and the intra-assay coefficients of variation were 5.17% (16.72 μmol/L) and 5.37% (8.86 μmol/L) for serum copper. The measuring methods and data reliability of potential confounders (e.g., serum iron, calcium, magnesium and phosphorus) are shown in Additional file 1.

### Assessment of radiographic knee chondrocalcinosis

Two orthopaedic surgeons were engaged to evaluate all the X-ray films while remaining blinded to the clinical and biochemical findings of corresponding subjects. Radiographic chondrocalcinosis was defined by evidence of knee joint linear cartilage calcification [31]. Specifically, prior to the assessment, the two orthopaedic surgeons were requested to review 200 X-rays films from the Osteoarthritis Initiative (OAI) for calibration purpose until they had agreed with each other on all these readings to a high level (the cutoff of simple kappa for the inter-rater reliability was 0.70). Then, the formal reading process started by reading each batch of knee X-rays in a mixed manner (100 X-rays in one batch: 10 previously-read X-rays and 90 unread X-rays that were randomly selected). The unread radiographs were utilized to test the inter-rater reliability, while the previously-read radiographs were utilized to test the intra-rater reliability. Both surgeons were requested to evaluate all the X-rays, and the inconsistencies, if any, were resolved through discussion. The Kappa value of the X-ray reliability reading was 0.72 (95% CI 0.67–0.77) for the inter-evaluator and 0.76 (95% CI 0.67–0.85) for the assessor’s internal reliability [24].

### Statistical analysis

The continuous data were expressed in the form of mean ± standard deviation, and the categorical data were expressed in the form of proportion (percentage). According to the tertile distribution in the study population, the serum zinc and copper concentrations were classified into three categories (i.e., ≤ 12.1, 12.2–14.2, and ≥ 14.3 mmol/L for zinc; and ≤ 14.7, 14.8–17.5, and ≥ 17.6 mmol/L for copper). Cu/Zn ratio was also calculated, and then classified into three categories according to its tertile distribution (i.e., ≤ 1.07, 1.08–1.38, and ≥ 1.39). The generalized estimating equation [32] was used to investigate the association of each of the serum zinc, copper and Cu/Zn ratio categories with chondrocalcinosis, under adjustment of potential confounders (knee-specificity analysis). The binary distribution and the logit linkage of the PROC GENMOD program in SAS were used to calculate the odds ratio (OR) and the related 95% confidence interval (95% CI) of chondrocalcinosis among various serum zinc and copper and Cu/Zn ratio categories, with the lowest tertile of serum zinc or copper or Cu/Zn ratio being taken as the reference. Specifically, the confounders of age (40–49 years old, 50–59 years old, 60–69 years old, ≥ 70 years old), body mass index (BMI) (< 28, ≥ 28 kg/m^2^) and sex (male, female) were adjusted first. Then, each of the following covariates: serum iron (tertiles), serum calcium (tertiles), serum magnesium (tertiles), serum zinc (tertiles), serum copper (tertiles) and serum phosphorus (tertiles) was added into the regression model.

Subsequently, the dose-response relationships between the serum zinc and copper concentrations and Cu/Zn ratio with the prevalence of knee chondrocalcinosis were plotted by generalized additive models with spline regression [33].

## Results

A total of 12,362 subjects (7017 men and 5345 women) aged ≥40 years (average 52.1 ± 7.9 years) were included in the analysis. The prevalence of knee chondrocalcinosis in the entire sample was 1.2% (Table 1).

**Table 1:** Basic characteristics among 12,362 participants according to knee chondrocalcinosis status

	Chondrocalcinosis status		*P*
	Yes	No	
Number	153	12,209	–
Median serum zinc level (μmol/L)	12.4	13.1	–
Median serum copper level (μmol/L)	17.5	16.0	–
Median Cu/Zn ratio	1.45	1.21	–
Age (years)	61.7 ± 10.1	52.0 ± 7.8	< 0.001
40–49 (%)	15.0	45.2	
50–59 (%)	27.5	37.7	
60–69 (%)	35.3	14.5	
≥ 70 (%)	22.2	2.6	
Gender (% female)	45.8	43.2	0.528
BMI (kg/m^2^)	24.9 ± 3.5	24.5 ± 4.0	0.161
< 28 kg/m^2^ (%)	83.0	87.0	
≥ 28 kg/m^2^ (%)	17.0	13.0	
Serum iron (μmol/l)	16.7 ± 5.2	18.5 ± 6.5	< 0.001
Serum calcium (mmol/l)	2.4 ± 0.1	2.4 ± 0.1	0.609
Serum magnesium (mmol/l)	0.88 ± 0.12	0.89 ± 0.08	0.023
Serum phosphorus (mmol/l)	1.1 ± 0.2	1.2 ± 0.2	0.840

*BMI* body mass index, *Cu/Zn ratio* the ratio of serum copper to zinc concentrations

Data are mean ± standard deviation, unless otherwise indicated

**Fig. 1:**
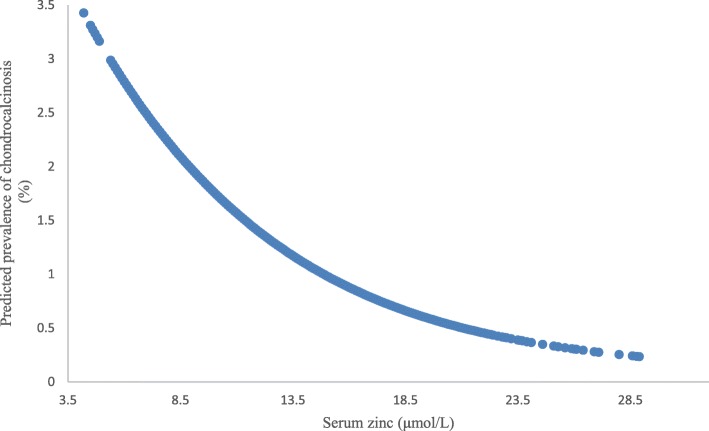
Association between serum zinc and predictive prevalence of chondrocalcinosis

Figure 1 shows the linear association between the serum zinc and predicted prevalence of chondrocalcinosis. Compared with the lowest tertile of serum zinc, the prevalence of knee chondrocalcinosis decreased by 0.5% in the middle and 0.9% in the highest. As shown in model 1, the ORs (95% CIs) for knee chondrocalcinosis, with adjustment for age, sex and BMI, were 0.74 (95% CI 0.50–1.09) and 0.56 (95% CI 0.36–0.86) from the second to the highest tertile of serum zinc, respectively, compared with the lowest tertile (*P* for trend = 0.009). With further adjustment for serum iron (model 2), calcium (model 3), magnesium (model 4), copper (model 5) and phosphorus (model 6) on the basis of model 1, the results were not substantially altered (Table 2).

**Table 2:** Association between serum zinc and knee chondrocalcinosis (*n* = 12,362)

	Tertiles of serum zinc (μmol/L)			*P* for trend
	1 (≤ 12.1)	2 (12.2–14.2)	3 (≥ 14.3)	
Total				
Knee chondrocalcinosis (%)	1.7	1.2	0.8	–
N for knee^a^	8354	8285	8082	–
Model 1 (95% CI)	1.00 (reference)	0.74 (0.50, 1.09)	0.56 (0.36, 0.86)	0.009
Model 2 (95% CI)	1.00 (reference)	0.77 (0.52, 1.14)	0.59 (0.38, 0.91)	0.018
Model 3 (95% CI)	1.00 (reference)	0.73 (0.49, 1.08)	0.54 (0.35, 0.84)	0.006
Model 4 (95% CI)	1.00 (reference)	0.75 (0.51, 1.12)	0.56 (0.37, 0.87)	0.010
Model 5 (95% CI)	1.00 (reference)	0.75 (0.51, 1.11)	0.58 (0.37, 0.88)	0.012
Model 6 (95% CI)	1.00 (reference)	0.75 (0.50, 1.11)	0.56 (0.37, 0.87)	0.011

Model 1 included age (40–49, 50–59, 60–69, ≥ 70 years), body mass index (< 28, ≥ 28 kg/m^2^) and sex (*n* = 12,362);

Model 2 added serum iron (tertiles) on the basis of model 1 (*n* = 12,357);

Model 3 added serum calcium (tertiles) on the basis of model 1 (*n* = 12,264);

Model 4 added serum magnesium (tertiles) on the basis of model 1 (*n* = 12,362);

Model 5 added serum copper (tertiles) on the basis of model 1 (*n* = 12,362);

Model 6 added serum phosphorus (tertiles) on the basis of model 1 (*n* = 12,264)

^a^Three right knees with K-L 4 grade were excluded for analysis (data from the contralateral knees were retained)

*N* number, *CI* confidence interval

**Fig. 2:**
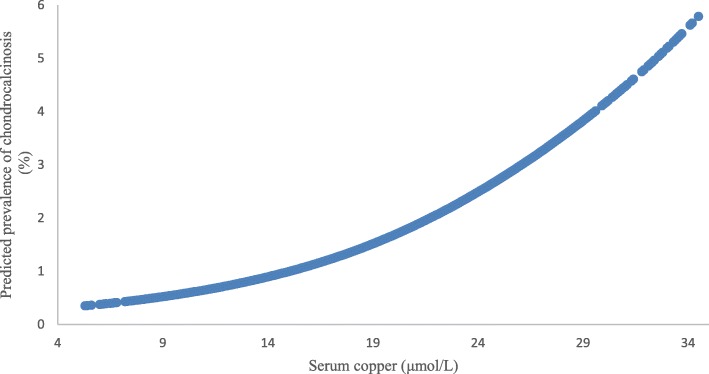
Association between serum copper and predictive prevalence of chondrocalcinosis

The dose-response relationship between the serum copper and predicted prevalence of chondrocalcinosis is shown in Fig. 2. The prevalence of knee chondrocalcinosis was 0.8% in the lowest tertile of serum copper and increased by 0.3% in the middle and 1.1% in the highest. The age, sex and BMI adjusted ORs (95% CIs) for knee chondrocalcinosis were 1.26 (95% CI 0.77–2.05) and 2.01 (95% CI 1.25–3.24) from the second to the highest tertile of serum copper, respectively, compared with the lowest tertile (*P* for trend = 0.003) (model 1). The results were not materially changed after further adjustment for potential confounders on the basis of model 1 (Table 3).

**Table 3:** Association between serum copper and knee chondrocalcinosis (*n* = 12,362)

	Tertiles of serum copper (μmol/L)			*P* for trend
	1 (≤ 14.7)	2 (14.8–17.5)	3 (≥ 17.6)	
Total				
Knee chondrocalcinosis (%)	0.8	1.1	1.9	–
N for knee*	8390	8190	8141	–
Model 1 (95% CI)	1.00 (reference)	1.26 (0.77, 2.05)	2.01 (1.25, 3.24)	0.003
Model 2 (95% CI)	1.00 (reference)	1.26 (0.78, 2.06)	1.94 (1.21, 3.12)	0.004
Model 3 (95% CI)	1.00 (reference)	1.24 (0.76, 2.02)	2.00 (1.24, 3.23)	0.003
Model 4 (95% CI)	1.00 (reference)	1.27 (0.78, 2.07)	2.11 (1.31, 3.38)	0.001
Model 5 (95% CI)	1.00 (reference)	1.22 (0.75, 1.99)	1.95 (1.21, 3.12)	0.004
Model 6 (95% CI)	1.00 (reference)	1.24 (0.76, 2.02)	2.00 (1.24, 3.22)	0.003

Model 1 included age (40–49, 50–59, 60–69, ≥ 70 years), body mass index (< 28, ≥ 28 kg/m^2^) and sex (*n* = 12,362);

Model 2 added serum iron (tertiles) on the basis of model 1 (*n* = 12,357);

Model 3 added serum calcium (tertiles) on the basis of model 1 (*n* = 12,264);

Model 4 added serum magnesium (tertiles) on the basis of model 1 (*n* = 12,362);

Model 5 added serum zinc (tertiles) on the basis of model 1 (*n* = 12,362);

Model 6 added serum phosphorus (tertiles) on the basis of model 1 (*n* = 12,264)

*Three right knees with K-L 4 grade were excluded for analysis (data from the contralateral knees were retained)

N, number; CI, confidence interval

**Fig. 3:**
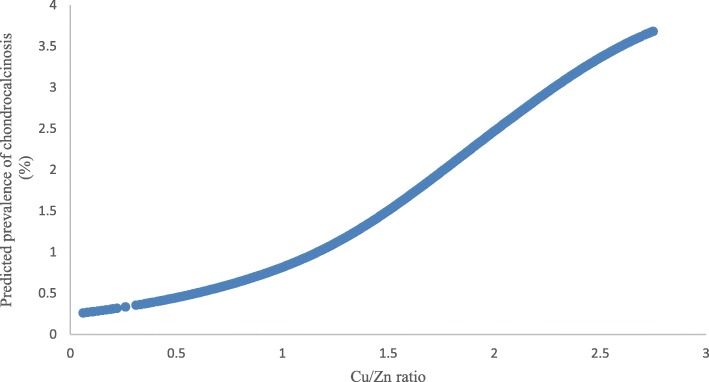
Association between Cu/Zn ratio and predictive prevalence of chondrocalcinosis. Cu/Zn ratio, the ratio of serum copper to zinc concentrations

Figure 3 illustrates the dose-response relationship between Cu/Zn ratio and predicted prevalence of chondrocalcinosis. A positive association between Cu/Zn ratio and the prevalence of chondrocalcinosis is illustrated in Table 4. The results did not change substantially after adjustment for potential confounders (i.e., age, sex, BMI, serum iron, serum calcium, serum magnesium and serum phosphorus).

**Table 4:** Association between Cu/Zn ratio and knee chondrocalcinosis (*n* = 12,362)

	Tertiles of Cu/Zn ratio			*P* for trend
	1 (≤ 1.07)	2 (1.08–1.38)	3 (≥ 1.39)	
Total				
Knee chondrocalcinosis (%)	0.7	1.0	2.1	–
N for knee^a^	8158	8406	8157	–
Model 1 (95% CI)	1.00 (reference)	1.02 (0.61, 1.69)	2.23 (1.38, 3.59)	< 0.001
Model 2 (95% CI)	1.00 (reference)	1.00 (0.60, 1.67)	2.12 (1.31, 3.42)	< 0.001
Model 3 (95% CI)	1.00 (reference)	1.03 (0.62, 1.72)	2.27 (1.40, 3.68)	< 0.001
Model 4 (95% CI)	1.00 (reference)	1.03 (0.62, 1.71)	2.26 (1.41, 3.63)	< 0.001
Model 5 (95% CI)	1.00 (reference)	1.01 (0.61, 1.68)	2.20 (1.36, 3.55)	< 0.001

Model 1 included age (40–49, 50–59, 60–69, ≥ 70 years), body mass index (< 28, ≥ 28 kg/m^2^) and sex (*n* = 12,362);

Model 2 added serum iron (tertiles) on the basis of model 1 (*n* = 12,357);

Model 3 added serum calcium (tertiles) on the basis of model 1 (*n* = 12,264);

Model 4 added serum magnesium (tertiles) on the basis of model 1 (*n* = 12,362);

Model 5 added serum phosphorus (tertiles) on the basis of model 1 (*n* = 12,264)

^a^Three right knees with K-L 4 grade were excluded for analysis (data from the contralateral knees were retained)

*N* number, *CI* confidence interval, *Cu/Zn ratio* the ratio of serum copper to zinc concentrations

## Discussion

To our best knowledge, this is the first study revealing positive associations between the serum copper, Cu/Zn ratio and the prevalence of chondrocalcinosis, as well as an inverse association between the serum zinc and the prevalence of chondrocalcinosis in contrast. Our findings are independent of some major confounders, such as age, sex, BMI, iron, calcium, magnesium, copper, zinc, and phosphorus, suggesting that the observed associations are robust.

While the biological mechanisms of linking the concentrations of serum zinc and copper to the risk of knee chondrocalcinosis are not fully understood, the inflammatory response may partly explain these findings. Zinc deficiency can lead to a proinflammatory status [13]. Previous studies have shown that a lower level of zinc concentration could reduce the natural killer cell immune activity, alter the inflammatory cytokine production, and impair the function of superoxide dismutase which would in turn alleviate the inflammatory response by converting O_2_^−^ free radicals into O_2_ and H_2_O_2_ [34–38]. On the contrary, a higher level of copper concentration could accelerate the catalytic action of H_2_O_2_ into hydroxyl radicals, which would then strengthen the reactive oxygen species-dependent killing in macrophages and thereby aggravate the inflammatory response [39].

The deposition of calcium pyrophosphate crystals on articular fibrocartilage or hyaline cartilage is one of the most typical features of chondrocalcinosis, and it can mediate tissue damage through the inflammatory mechanism [1, 2]. Extracellular trap release can be increased in human neutrophils activated by microcrystals, and the NALP3 inflammasome is induced once the calcium pyrophosphate crystals are deposited [9, 22]. In addition, calcium pyrophosphate crystals can directly catabolize chondrocytes and synoviocytes, resulting in the production of destructive matrix metalloproteinases and prostaglandins, which are both of great importance in the process of inflammatory response [23, 40]. Thus, the inflammatory response may explain, at least in part, the significant associations between the serum zinc and copper concentrations and the prevalence of chondrocalcinosis.

Cu/Zn ratio reflects the balance between copper and zinc in the human body. Rather than a simple nutritional indicator, previous studies showed that it could also be a potential biomarker of inflammatory response and oxidative stress [21, 41]. Moreover, it has been reported to be positively correlated with disability and mortality in elderly subjects aged 70 years and above [21]. The present study showed that lower Cu/Zn ratio is associated with a lower prevalence of knee chondrocalcinosis, which also assessed the robustness of our findings of the associations between serum zinc and copper concentrations and the prevalence of chondrocalcinosis, to some extent.

Several advantages of this study are noteworthy. First of all, a large sample size (*n* = 12,362) of this first study investigating the association between serum zinc and copper concentrations and Cu/Zn ratio and knee chondrocalcinosis guaranteed that our findings are likely to be of a low occasionality. Second, the serum zinc, copper concentrations and Cu/Zn ratio are respectively correlated with the prevalence of chondrocalcinosis in a dose-response manner. This observation has important implications for both zinc and copper as trace elements and for the disease of chondrocalcinosis itself, suggesting that there may be correlations between these two elements and the pathogenesis of chondrocalcinosis.

However, this study is also involved with several caveats. First, we cannot draw a causal relationship between the serum zinc and copper concentrations and Cu/Zn ratio and the prevalence of chondrocalcinosis due to the cross-sectional design. Nevertheless, as no previous research has investigated the associations between these two elements and chondrocalcinosis, the value of this study should not be diminished. Second, other views of the knee (such as lateral or skyline) were not used to in our study ascertain radiographic chondrocalcinosis; thus, the prevalence of radiographic chondrocalcinosis might be underestimated. Third, the synovial fluid (taken from the joint) test should be more sensitive and specific than radiographs for detecting crystals, and some of the articular calcium pyrophosphate depositions may be too small to be detected on radiographs; thus, our estimation of the prevalence of knee chondrocalcinosis is likely to be underestimated.

## Conclusions

The present study observed that higher serum zinc concentrations, lower serum copper concentrations or lower Cu/Zn ratio are associated with a lower prevalence of knee chondrocalcinosis in a dose-response relationship manner.

## Supplementary information

**Additional file1.** Measuring methods and reliability data of potential confounders.

## Data Availability

Data can be requested from the corresponding author.

## Abbreviations

3,5-diBr-PAESA: 4-(3,5-dibromo-2-pyridylazo)-N-ethyl-N-(3-sulfopropyl) aniline
5-Br-PAPS: 2-(5-Bromo-2-pyridylazo)-5-[N-propyl-N-(3-sulfopropyl) amino] phenol
BMI: Body mass index
CI: Confidence interval
Cu/Zn ratio: The ratio of serum copper to zinc concentrations.
KL: Kellgren-Lawrence
OAI: Osteoarthritis Initiative
OR: Odds ratio
