# Supplementary material for: Association between serum zinc and copper concentrations and copper/zinc ratio with the prevalence of knee chondrocalcinosis: a cross-sectional study
Source: BMC Musculoskelet Disord. 2020 Feb 12;21:97. doi: 10.1186/s12891-020-3121-z (PMC7017625; doi:10.1186/s12891-020-3121-z)
Supplement: Supplementary file 1 — Additional file1. Measuring methods and reliability data of potential confounders. [file 12891_2020_3121_MOESM1_ESM.docx]

**Measuring methods and reliability data of potential confounders**

**Serum iron**

The serum iron concentration was measured using the Ferene method by Roche modular P800. For serum iron, the inter-assay coefficients of variation were 1.426% (11.6 umol/L) and 0.6% (44.5 umol/L), and the intra-assay coefficients of variation were 1.55% (43.95 umol/L) and 1.03% (11.29 umol/L).

**Serum calcium**

The serum calcium concentration was measured using the Arsenazo III method by Beckman Coulter AU 5800 (Beckman Coulter Inc., Brea, CA, USA). For serum calcium, the inter-assay coefficients of variation were 1.03% (3.01 mmol/L) and 0.86% (2.33 mmol/L), and the intra-assay coefficients of variation were 0.86% (2.35 mmol/L) and 0.58% (3.56 mmol/L).

**Serum magnesium**

The serum magnesium concentration was measured using the chemiluminescence method by Beckman Coulter AU 5800. For serum magnesium, the intra-assay coefficients of variation were 1.86% (0.60 mmol/L) and 1.65% (1.00 mmol/L), and the inter-assay coefficients of variation were 1.87% (0.60 mmol/L) and 1.70% (1.00 mmol/L).

**Serum phosphorus**

The serum phosphate concentration was measured using the phosphomolybdate method by Beckman Coulter AU 5800. For serum phosphate, the inter-assay coefficients of variation were 2.38% (2.48 mmol/L) and 2.41% (1.16 mmol/L), and the intra-assay coefficients of variation were 0.66% (1.12 mmol/L) and 0.33% (2.84 mmol/L).
